# Turnover Intention Among Direct Care Workers of Older Adults in Chinese Hospitals and Nursing Homes

**DOI:** 10.1093/geroni/igaa057.284

**Published:** 2020-12-16

**Authors:** Zhenzhen Zhang, Shuangshuang Wang, Nengliang (Aaron) Yao, Zhang Zhenzhen

**Affiliations:** Shandong University, Jinan, Shandong, China

## Abstract

High retention rates among direct care workers (DCWs) affect the quality of aged care. However, limited research has explored factors associated with retention in the Chinese aged care industry. This study compared turnover intention and job satisfaction among DCWs in Chinese hospitals and nursing homes. A total 370 DCWs from 7 hospitals (297 contractual, 73 non-contractual) and 311 DCWs from 7 nursing homes (27 contractual, 284 non-contractual) located in Fujian, China were recruited to fill out a questionnaire. Overall, DCWs from hospitals reported lower turnover intention (20.5 % vs 37.0%) and higher levels of job satisfaction (31.1% vs 16.4%) than DCWs from nursing homes. Specifically, contractual DCWs from hospitals indicated lower turnover intention (14.8%) than non-contractual DCWs from hospitals (43.8%) and both types of DCWs from nursing homes (36.3% and 44.4%). Higher job satisfaction was associated with lower turnover intention, but did not mediate the association between DCW types and turnover intention. Findings suggested that the government and institutions should help DCWs complete the identity transformation from non-contractual DCWs to contractual DCWs to enhance job security and benefits. For nursing home DCWs, licensing and registration requirements shall meet the standards for hospital DCWs. Attention is also to be paid to working conditions and staff welfare of DCWs, including social insurance, pensions, and trainings, to improve job satisfaction and reduce turnover intention.

